# Nurturing innovative culture in a healthcare organisation – Lessons from a Swedish case study

**DOI:** 10.1108/JHOM-05-2021-0181

**Published:** 2023-01-24

**Authors:** Thomas Andersson, Gary Linnéusson, Maria Holmén, Anna Kjellsdotter

**Affiliations:** School of Business , University of Skövde , Skövde, Sweden; Innovation Platform, Region Västra Götaland , Gothenburg, Sweden; Research and Development Centre , Skaraborg Hospital , Skövde, Sweden; School of Engineering, Jönköping University , Jönköping, Sweden; Faculty of Theology, Diaconia and Leadership, VID Specialized University , Oslo, Norway

**Keywords:** Innovation, Healthcare organisation, Organisational culture, Leadership, Innovative culture, Value

## Abstract

**Purpose:**

Healthcare organisations are often described as less innovative than other organisations, since organisational culture works against innovations. In this paper, the authors ask whether it has to be that way or whether is possible to nurture an innovative culture in a healthcare organisation. The aim of this paper is to describe and analyse nurturing an innovative culture within a healthcare organisation and how culture can support innovations in such a healthcare organisation.

**Design/methodology/approach:**

Based on a qualitative case study of a healthcare unit that changed, within a few years, from having no innovations to repeatedly generating innovations, the authors describe important aspects of how innovative culture can be nurtured in healthcare. Data were analysed using inductive and deductive analysis steps.

**Findings:**

The study shows that it is possible to nurture an innovative culture in a healthcare organisation. Relationships and competences beyond healthcare, empowering structures and signalling the importance of innovation work with resources all proved to be important. All are aspects that a manager can influence. In this case, the manager's role in nurturing innovative culture was very important.

**Practical implications:**

This study highlights that an innovative culture can be nurtured in healthcare organisations and that managers can play a key role in such a process.

**Originality/value:**

The paper describes and analyses an innovative culture in a healthcare unit and identifies important conditions and strategies for nurturing innovative culture in healthcare organisations.

## Introduction

There is a need for innovation in healthcare to balance cost containment and healthcare quality, as well as to meet the needs of its different stakeholders ( Larisch *et al.* , 2016 ; Gomes Chaves *et al.* , 2021 ). Considering increasing healthcare demands, resources will simply not be enough to provide sufficient care if healthcare does not innovate ( Gadolin and Andersson, 2017 ). However, despite the understanding that innovation is a critical capability of healthcare organisations ( Savory and Fortune, 2015 ), research has been surprisingly uninterested in the subject in public healthcare compared to other sectors ( Arya, 2016 ). Furthermore, the active role of healthcare organisations in generating innovations is often underestimated in research ( Thune and Mina, 2016 ). Instead, healthcare organisations are either portrayed as passive consumers/implementers of innovations that appear outside the organisation (e.g. Cranfield *et al.* , 2015 ), or they are only visible as “background” when research focuses on innovative physicians ( Bosa, 2008 ) or entrepreneurs ( Exton, 2008 ). In line with Thune and Mina (2016) , we argue that there is a need for more knowledge on the active role of healthcare organisations in innovation processes and how they can contribute to innovations. Similarly, Øvretveit *et al.* (2012) argued that there are good reasons to criticise the dominant implementation view on innovation in healthcare, since it indicates that healthcare organisations are passive receivers. As a response to this myopic view, Øvretveit *et al.* suggested the concept of “developmental evolution”, as it better describes change processes related to innovations than the “implementation view” does. First, developmental evolution as a concept questions the extent to which such processes could be rationally planned. Second, the concept views healthcare organisations as having an active function in such change processes. The dominant view of healthcare organisations as passive implementers ( Thune and Mina, 2016 ) gives the impression that innovation is performed elsewhere than within healthcare organisations, which take away incremental and local aspects of innovation processes ( Jurado-Salgado *et al.* , 2022 ). In this paper, the focus is not on more radical innovations ( Jurado-Salgado *et al.* , 2022 ), but on innovation processes that are incremental where a healthcare organisation is more likely to have an active role. In short, we see innovation as an intended organisational change to produce better outcomes that contrast with how things are currently done ( Osborne and Brown, 2011 ; Arya, 2016 ), and the focus of this paper is on incremental innovation processes, where healthcare organisations are active parts.

When viewing healthcare organisations as an active part in innovation processes, research has identified organisational culture as one of the most important antecedents of healthcare organisations' innovativeness ( Acar and Acar, 2012 ; Thakur *et al.* , 2012 ; Kash *et al.* , 2014 ; Arya, 2016 ; Kelly and Young, 2017 ; Day-Duro *et al.* , 2020 ). However, since a majority of studies have used quantitative methods ( Länsisalmi *et al.* , 2006 ; Thakur *et al.* , 2012 ), research tends to state that organisational culture is important for healthcare innovation rather than how it is important. Furthermore, organisational culture is most often treated as a current state (e.g. Do Carmo Caccia-Bava *et al.* , 2006 ; Acar and Acar, 2012 ) rather than as something processual that is evolving and can be nurtured (e.g. Lindblad *et al.* , 2017 ; Day-Duro *et al.* , 2020 ). In other words, the dominant view of organisational culture in healthcare research is that it is something static that the organisation has, rather than as something processual that describes what the organisation is (cf. Smircich, 1983 ) and its direction of becoming. Consequently, there is a need for more research that describes how culture as a process can support innovations in healthcare. This point is underscored by the fact that organisational culture is not only identified as an important condition for healthcare innovations, but also as one of the possible main blocking mechanisms for innovations ( Wycoff, 2003 ; Keller *et al.* , 2013 ; Mannion and Davies, 2018 ). Since organisational culture can be understood as the set of meanings and values that members of the organisation share, it governs individual actions by defining appropriate ways to think and act with regard to the organisation ( Watson, 2001 ). Accordingly, it can be logically derived that organisational culture can both nurture innovation and block innovation, depending on whether dominant values either support innovation or not. The questions are, how can an innovative culture be nurtured and how can it influence the innovativeness in a healthcare organisation? Therefore, the aim of this paper is to describe and analyse the nurturing of an innovative culture within a healthcare organisation and ask how culture can support innovations in such a healthcare organisation.

When using organisational culture as a concept in healthcare organisations, it is important to understand the possible misalignment of the concept in this context. Organisational culture focuses on shared beliefs in an organisation, but the question is, to what extent are there shared beliefs in a healthcare organisation? First, sub-cultural diversity within healthcare organisations is often over-looked in favour of organisational-level assessments of cultural characteristics ( Scott *et al.* , 2003 ). Second, healthcare professionals tend to commit more to professional values than organisational values ( Wallace, 1995 ), where professional cultures often have a stronger influence on professionals' actions than organisational culture in healthcare ( Gadolin and Andersson, 2017 ). However, this does not mean that professional culture and organisational culture are separate; instead, they become increasingly intertwined. Since professionals perform their work in organisational settings, organisational aspects have become increasingly important in order to understand professions ( Liff and Andersson, 2011 ; Muzio *et al.* , 2013 ). Third, the degree of innovativeness can also differ within one healthcare organisation based on different (sub-) cultural characteristics that support innovation, to varying extents ( Exton, 2010 ; Llopis and D’Este, 2016 ). Fourth, the influence of culture on managers' and professionals' identities may also vary ( Andersson, 2015 ). Finally, culture gives power positions to certain actors, such as physicians in healthcare ( Scott, 2008 ), by indicating what and who is perceived as important. Therefore, these actors may have the best possibilities to generate change, but may also be the ones who are the least interested in actually changing current institutional order ( Andersson and Gadolin, 2020 ), since it may threaten their power position. In sum, there are many difficulties related to using organisational culture to explain innovativeness in healthcare organisations, but Exton (2010) argued that this should not prevent researchers from using organisational culture as concept; rather, it makes it even more important to understand good examples. The context matters and qualitative case studies could increase the understanding of what matters. Accordingly, in the present study we have chosen to perform a qualitative case study of a healthcare organisation that has been very successful (successful here means generating many innovations consistently over time) in nurturing an innovation culture to contribute to the understanding of how organisational culture can support innovation in healthcare organisations.

## Previous research on innovative culture in healthcare organisations

Many studies on healthcare innovation have an individual focus, where the importance of entrepreneurial experimentation among these individuals is at the centre of the description (e.g. Exton, 2008 , 2010 ). However, despite their individual focus, these studies also describe how organisational aspects influence the behaviour of these individuals. They show how healthcare culture in general does *not* support innovation ( Cinar *et al.* , 2019 ), by illustrating how the healthcare organisational structure and culture often focuses on conformity, consistency, efficiency and survival ( Arya, 2016 ), which is contrary to innovation and novelty ( Dopson *et al.* , 2008 ). In such conditions, initiatives for innovation will most often be resisted, even if individuals are entrepreneurial ( Exton, 2010 ). However, organisational culture in healthcare should perhaps not support innovation at any price, since matters like patient safety culture ( Vogus *et al.* , 2010 ; Brandis *et al.* , 2017 ) are prioritised cultural aspects in healthcare. Patient safety culture is and should be important in healthcare organisations, which will inevitably mean that experimentation and entrepreneurial action is somewhat delimited.

An empowering organisational culture is presented as central to support innovation in healthcare organisations ( Kelly and Young, 2017 ). Since there is a structural dimension of empowerment, where organisational structure provides power, organisational structure is intertwined with culture when it comes to empowerment ( Andersson *et al.* , 2022 ). Structure and culture are often treated as separate subjects, although, in practice, they are intertwined and interdependent in their influence of each other ( Watson, 2001 ). Furthermore, when organisational structure constitutes manifestations of certain values, it can be considered an aspect of organisational culture ( Andersson *et al.* , 2019 ). Exton (2010) aligned with the empowerment argument and described how the organisational structure can support individuals in entrepreneurial actions, such as whether innovation is sanctioned and legitimate at all managerial levels, when individuals in the organisation have autonomy, and when there are opportunities for creativity between organisational boundaries. It would then mean that an organisational culture that supports innovation in healthcare is characterised by managers who legitimate and value innovation, autonomy that enables people to think beyond their existing ways of working and the appreciation of meeting other competencies when working across organisational boundaries.

While managers are obviously important for influencing a certain culture, Øvretveit *et al.* (2012) also described how clinical leaders are even more important than senior managers in healthcare. Clinicians have been considered more dominant than other actors when it comes to influence values in healthcare organisations ( Scott, 2008 ). Exton (2008) further emphasised that if healthcare managers should be the entrepreneurs for development and innovation in healthcare, their identities would need to develop towards entrepreneurial orientation rather than the current regulatory structures. However, New Public Management has pushed healthcare organisations towards an increased regulatory focus and quantitative measuring rather than towards entrepreneurial orientation ( Eriksson *et al.* , 2020 ). Culture is often described as providing identity templates that individuals can elaborate on in their identity processes ( Watson, 2008 ), but what Exton (2008) showed is that the identity templates that healthcare culture provides direct managers' identity processes towards regulation rather than entrepreneurial orientation. Regardless of whether it is managers ( Exton, 2008 , 2010 ) or clinical leaders ( Øvretveit *et al.* , 2012 ) that are seen as the most important bearers of values that support innovation, Larisch *et al.* (2016) described lack of leadership as one of the main reasons why healthcare organisations give limited attention to innovation. Since leadership in terms of culture can be considered providing direction and ways to think, prioritise and define what is valued, leadership does not seem to direct attention to innovation in healthcare organisations.

Previous research provides some important examples that describe organisational culture as something that could nurture innovation in healthcare organisations. Day-Duro *et al.* (2020) emphasised that organisational culture in healthcare can be developed to support innovation, but that it is then important to manifest values that people find important. They empirically described how a culture of respect, valuing and investing in individuals, can facilitate successful innovation. They further emphasised the importance of leadership in nurturing such a culture, by balancing between kindness and empowerment that allows people to act, and manifesting conviction in a vision to create direction, but also requiring accountability to direct empowered actions. Kelly and Young (2017) also emphasised a culture of empowerment as cultural characteristic to support innovation in healthcare organisations. This confirms Exton's (2010) description of how autonomy is important for innovations, but also complements that organisational culture may provide frames for such autonomy. Day-Duro *et al* . (2020) also concluded that since values become shared through personal interactions, it is important to provide opportunities to meet and form personal and professional connections in healthcare organisations to support innovation.

To sum up, previous research has mainly described how organisational culture in healthcare does *not* tend to support innovation, but that there are great variations in innovativeness between different units in healthcare and that it is possible to learn from the good examples. This study will address this call to learn from good examples.

## Theoretical framework for analysing culture

Organisational culture is an important concept because it governs people's actions in an organisation based on shared assumptions and shared values among its members ( Watson, 2006 ). When we describe an innovative culture as in this article, we do not see innovative culture as something separate; rather, it is the part of the organisational culture that supports innovation by governing people's actions in such direction.

The patterns of values, beliefs and basic assumptions that make organisational culture are not “things” that can be easily understood ( Watson, 2006 ). However, there are cultural aspects that we can observe, like things, behaviour and talk, but it is not self-evident that we understand what they mean. The most common model to analyse organisational culture is based on Schein's (1990 , 2017) writing, in which he emphasised that culture is partly visible and partly invisible. This is illustrated by the Iceberg model containing Schein's (1990 , 2017) three different levels of culture, with the term level meaning the degree to which a cultural phenomenon is visible. The first level, *artefacts* , is above sea level and visible for an observer, whereas the two other levels, *espoused values* and *basic assumptions* , are below the water in the Iceberg model; this illustrates that they are not visible for an observer but are manifested (and thereby implicitly observable) through the *artefacts* level. The cultural essence is represented by the *basic assumptions* , which are deeply embedded and unconscious; this also means that they have the strongest influence on people's behaviour, because people take them for granted and do not really see the alternatives. *Espoused values* , beliefs, norms and rules connect the *basic assumptions* and the tangible manifestations on the *artefacts* level.

Consequently, analysing organisational culture requires delving below the visible artefacts level, which constitutes manifestations of the culture rather than the culture itself, to reach the less visible layers of culture ( Watson, 2006 ). Artefacts can be physical things, behaviour and language that are observable, but it is not self-evident which values they symbolise. Thus, a cultural analysis means analysing what values these artefacts symbolise ( Watson, 2006 ); what they say about what is perceived as important/not important, good/bad, etc. Through these values we can analyse which basic assumptions about, for example, time and people these values and artefacts are based upon. In the present case, the cultural analysis will be directed towards how culture supports innovation.

## Method

### Case and settings

The setting for the case study is a medium-sized county hospital that is located in the south-west part of Sweden and has about 30 medical specialties distributed in four geographical locations. The catchment area has approximately 270,000 inhabitants. The speech therapy unit was chosen as a case to understand how culture can support innovation, as well as how such culture could be nurtured. The case was a part of a larger multi-case research programme, the purpose of which was to map and analyse the innovation system in and around a hospital. During the initial data collection process in the overall programme, this unit stood out as an interesting case to better understand how an innovative culture could be nurtured, since the unit had produced a continuous flow of innovations in recent years, whereas they had not produced any in earlier years. There were rather pragmatic considerations that led to this particular case. This stood out in the overall mapping of cases in the multi-case programme as one that appeared to offer something to learn about the organisational aspects of innovation in healthcare and how these could be nurtured since: (1) the unit had had a continuous flow of innovations that was not connected to a single person in the unit, but a number of different people were involved in different innovation processes; (2) there seemed to have been some sort of change in the approach, since the unit historically not had stood out as particularly innovative; and (3) the initial interviews indicated that it was a certain way of thinking about their activities and work (what would later lead to a theoretical framework on organisational culture) that led to the innovations.

The case – the speech therapy unit – is available in two locations and treats people with speech and language difficulties, reading and writing difficulties, voice problems, stuttering, and difficulty eating and swallowing. Furthermore, the speech therapy unit receives referrals from the healthcare centre, child healthcare, school healthcare, other care providers or through a self-care referral. In addition to treatment at the unit, various support and treatment programmes are offered via the Internet, as well as digital care meetings with care providers. These supports and programmes have been a result of several innovation projects in recent years, especially regarding support and treatment through digital channels. With the help of innovation grants, several different pilots have been started at the unit, resulting in a continuous flow of innovations at the unit. The case unit also seemed to have evolved its organisational culture to better support innovation.

### Study design and sample

A qualitative single case study approach was chosen because of its value in analysing complex events with the capacity to obtain in-depth information about the topic ( George and Bennett, 2005 ; Polit and Beck, 2021 ). Semi-structured interviews were conducted with 14 informants (13 women and one man) who represented various professionals, mainly speech therapists, but also unit manager and academics. When choosing interviewees, we looked for interviewees who had been active participants in innovation projects and also those who had not participated, since we wanted to capture the culture rather than certain innovative individuals.

### Data collection

Methodologically, an open-ended qualitative interview seemed to be an appropriate data collection method. The interviews were semi-structured around a few main open questions in order to leave the participants free to narrate about their experiences and specific examples of innovative working and collaboration. Since the interviews aimed to describe processes, questions were posed in a way that invited the interviewees' response to be more narrative. The interview guide was developed by all the authors, including questions such as: “Can you please tell us about yourself?”, “Can you please describe processes you have experienced with working with innovation processes?”, “What role have you played in the organisation and what is your role today?”, and “Can you describe the process of different innovation project from the beginning to where they are today”. All four authors participated in interviews and there were always at least two interviewers in every interview. Continuous discussions among the interviewers were performed before and between the interviews to continuously analyse the material and find subjects to develop in coming interviews. The participants were asked to reflect on and describe their experiences of key roles and skills for the enablement of the innovation project, as well as obstacles identified by clinical staff to be able to manage a project within the hospital environment.

At the time of the initial contact by e-mail and telephone, participants were informed of the purpose of the study and the conditions of participation and given guarantees of confidentiality. Four interviews took place at the hospital where the interviewees could break away from ordinary hospital clinical activity. The other interviews were conducted digitally face-to-face (due to the COVID-19 pandemic). Data were collected between March 2020 and December 2020. In total, 14 interviews were performed. They were audio-recorded and lasted for between 26 and 83 min each (the average interview was around 60 min) and were transcribed verbatim.

### Data analysis

The interview texts were first analysed inductively, and were later analysed deductively using an analytical framework of organisational culture ( Schein, 1990 , 2017 ) that was chosen as a result of the first inductive analysis. The inductive analysis steps were based on thematic analysis, following Braun and Clarke (2006) , using six phases of thematic analysis: familiarise, generate initial codes, develop themes, review potential themes, define and name themes, and produce the report. In a first step, all the interviews were read several times as whole entities to obtain familiarity with all aspects of the data. The interviews were read by all authors individually and then discussed in the whole research group. Quotes that indicated relevant themes were highlighted in the text, and interpretations of what was in the data were written down. The second step involved identifying and coding meaningful units containing whole sentences or parts of text describing same content. The different codes were discussed by all authors together and then collated into potential themes. Finally, the themes were discussed and reviewed in relation to the coded units of text and the entire data set. In this stage, we had five main themes, all of which concerned shared values to different extents. We then turned to the analytical framework: Schein's (1990 , 2017) model of organisational culture. In a deductive analytical step, we used this analytical framework to analyse how different visible aspects of the culture (artefacts) related to espoused values. We further analysed how these manifested different basic assumptions. The artefacts and values served as structure of the results section, where the themes constitute headings. The results section describes the nurturing of an innovative culture, where the first sub-heading describes how the process of nurturing an innovative culture started, the three sub-headings in the middle describe the most important values identified, and the final sub-headings described the sustainability of the culture.

The analysis involved constantly moving between the entire data set, the coded meaningful units of the text and the on-going analysis of the data. During the entire analysis process, continuous discussions among the researchers were conducted to strengthen the consistency of the findings, as according to Polit and Beck (2021) . All authors have been active parts in all analytical steps. Quotations from the interviews are used to illustrate the content of the themes. The authors have different areas of expertise, which provides different perspectives and opportunities for analytical discussions of the findings. The findings are illustrated with appropriated quotes to enhance trustworthiness.

### Ethical considerations

The participants received oral information explaining the aim of the research, that participation was voluntary, that they could withdraw from the study at any time, and that the data would be treated confidentially.

## Results: Nurturing innovation culture in healthcare

The results section concentrates on the visible and partly visible layers of organisational culture – artefact level and espoused values – according to the previously presented analytical framework ( Schein, 1990 , 2017 ). The artefacts can be physical things, behaviour and language that are observable but it is not self-evident which values they symbolise. Thus, a cultural analysis involves analysing what values these artefacts symbolise ( Watson, 2006 ); what they say about what is perceived as important/not important, good/bad etc. These two layers are presented here in the result chapter, whereas the third layer – basic assumptions – require further analysis and is presented in the beginning of the discussion section.

### Nurturing an innovative culture – manager manifesting values

When talking about their work with innovation processes at the unit, the professionals most often described it in terms of change – that it has not been this way before, but now innovation work was part of their ordinary work.

It comes very natural today … it is a way of thinking … that we can change and develop the ways we work and meet patients. But it hasn’t always been like that; we used to do like most units in healthcare, solving problems by running faster. (Employee F)

When describing the starting point of this new approach to innovation, the professionals at the unit described their former unit manager as central in many ways for the current innovation climate. She had been important for nurturing the current culture at the unit, and the culture at the unit manifested the values that she was the bearer (and to some extent even the protector) of.

She walked her talk. She said that we could improve our activities, but she backed it up by directing us to people who could help us, applying for resources, letting us have time for innovation work. (Employee H)

The above quote shows that values need to be manifested repeatedly in order to be part of a culture. The manager had considerable influence on the dominant ideas and ways of thinking at the unit, and her actions can be considered to have manifested values that were important for the innovation culture. From a cultural view, the quote illustrated how the manager’s actions manifested certain values, but also that the employees appreciated the actions and the values they represented and aligned to these values. The employees described her as accessible and a good listener, which seemed important for influencing values to become shared.

In the following, we will describe these “new” values and how they were applied in practice, but also how “new” practices represented new values and nurtured the innovative culture. The values and artefacts we will describe are: (1) relationships and competence – beyond healthcare, (2) empowering structures and (3) resources signalling importance.

1. Relationships and competences – beyond healthcare

One part of the new approach to innovative work was the way the professionals at the unit thought about relationships with others and thereby, implicitly, the appreciation for competences beyond their own health professional competence. The former manager's external network was important, but employees also described how she generously shared her network with them. Acting in this manner created more of an external focus for the unit, almost as a business intelligence function, where people with other competences, such as digitalisation, improvement work and design thinking made it easier and more natural for the professionals to open their eyes for new ways of thinking and thereby new solutions and approaching problems in new ways. Some of these people/competences were external from the university, such as Information Technology (IT) researchers, whereas some were internal at the hospital but from other units, such as improvement workers.

I have several examples of people with other competences that really have influenced our ways of thinking, but the most striking example is when we had a workshop with a master student in computer gaming. It was a new world for us. It was so useful, we could apply this thinking directly on how we could think about making people repeat exercises they get. (Employee A)

The former manager encouraged and financed competence development beyond healthcare as means to enable innovation. Many of the professionals (including the manager) took courses on service design, which made them re-think their view of patients from not only caring for the patient, but also providing a service to a group of patients, and that service could (and should) be improved. One of the employees elaborated on how the course had influenced them:

Patients drive their individual interest. We realise that when we select improvements and innovations, we must think about groups of patients. The innovation should make it better for many patients, not only for one. (Employee E)

Employee E illustrated how she and her colleagues selected what to improve and what to direct their innovation work to. The service design education made them think about treatment not only as a treatment, but a service. They also realised that there is greater leverage in innovating a service with a large group of users than one single user.

In general, the examples above also illustrates how the employees had evolved in their approach to their work from “performing work within certain frames” to also “influencing the frames of work”, and also regarding their view on patients from “make the best for the individual patient here-and-now” to also consider “make it better for future (groups of) patients”. Furthermore, an integrated resource perspective was also present in these new ways of thinking, where innovations for the large groups of patients potentially released time for personnel to further improve care of individual patients who do not fit into the larger patient groups' needs. We return to this point under Theme 3.

1. Empowering structures

Regarding shaping the values towards increasing taking initiatives and including improvements as a natural part of work, it is important to mention the bottom-up perspective that the former manager advocated, which manifested in structures and activities. The former manager described herself as follows:

I used to say to the employees that it is okay to complain as long as you are willing to take initiative and do something about it. Then there is something constructive in complaints and frustration that enable innovations. (Former manager)

Three of the employees repeated this point independently of each other, noting that it was very important for the innovative culture. One of them elaborated as follows:

My perception is that moaning is typical for healthcare organisations. We love to moan, but we never do anything about problems. Here, this complaining lead to something constructive. And I have also learnt to not to complain if I am not willing to do anything about it (laughter). (Employee B)

The former manager worked actively to empower the employees, not only through structures, but also by nurturing how she wanted them to approach their work. She not only wanted the decentralised structure to allow them to take initiatives to improve, but also urged them to take initiatives to improve.

Today, we see innovation as part of our job, no one needs to tell us that. We just do it! (Employee, C)

Decentralisation was not only considered a structural aspect; it was just as much a part of the culture. The shared values that the structure and the manager's actions nurtured were an active approach to work among employees, where they saw themselves as active, initiative-taking, responsible and developing (see the quote above). This resulted in employees being involved in all parts of innovation work, from identifying needs of innovations to being designers of innovation and leading innovation projects. However, an important aspect of making the employees dare to take such steps was that they felt trusted by their manager, which was manifested in an open climate where it was legitimate to “think out loud” (Employee A) and try new ways of performing and organising work.

1. Resources signalling importance

As mentioned previously, resources were seen as central for the innovative climate at the unit. The current manager described how they viewed the relationship between resources and innovation:

Something we have learnt during the years is that innovation requires resources. We cannot expect innovation to just happen if we don’t provide resources for the employees to work with innovation projects. Innovation cannot be something you do on top of everything else you do; it must be a task in itself. (Current unit manager)

The speech therapy unit found resources for its innovation work both internally and externally. Externally, there was a newly started regional unit that supported innovation in healthcare in the region, from which the speech therapy unit applied and received funds for a specific innovation, which turned out to be successful for the unit. The success led to continued applications for funds for another project in the coming year, and it ended up as an evolving yearly process of applying for (and most often receiving) funds at the regional unit for innovation support. The funding was used to provide time for employees to work with innovation projects. However, the former manager also emphasised that there was a need for time to reflect in their daily work, not only in development projects. She also said that they would have focused on innovations independently of funding beyond their ordinary budget, but that processes would have been much slower without this funding.

Internally, the former manager received little support from managerial levels above. Instead, their focus on short-term cost made innovation work more difficult. Consequently, when talking about an innovation culture, it concerned this particular unit, not the hospital as a whole. It could even be said, frankly, that the innovation culture at the unit appeared *despite* the culture at the hospital, rather than because of it. The former manager's approach to this dilemma was to not openly apply for innovation work from her manager, but instead to find space within her ordinary budget to support innovative work:

I realised that it was no meaning to apply for funds for innovation in the budget process, because it would most likely be declined. I mean, they cheered our innovations, but they were not willing to pay for them. Instead, I found resources within my ordinary budget. But it never meant that I exceeded my budget, I found spaces within the budget to support innovation work. (Former manager)

Rationally, this approach improved conditions for innovations work, but in terms of culture it also symbolically manifested what was perceived as important at the unit.

One reflection that the professionals made regarding resources was that it might be easier to work with innovations in outpatient clinics than in wards with inpatient care. This is because, in terms of resources and time management, it is easier to plan their activities when they are not continuously interrupted by acute tasks.

It is easier when the patient is a name on a waiting list, compared to inpatients whose needs have to be met when they appear. It makes it possible to plan innovation work, which I think would be much more difficult in an acute unit. (Employee B)

The ability to plan one’s time better makes it easier to plan time for innovation work.

### Sustainable innovative culture

The interviewees described the nurturing of the innovative culture as a change from a less innovative culture to a more innovative culture at the unit. It also seemed as if the more innovative culture was not temporary, but that it seemed sustainable. There were several aspects that made the more innovative culture sustainable.

Internally, the decentralised structure and the manager's actions outlined above nurtured the employees' view of themselves as active and taking initiatives. Even if organisational structure and organisational culture are often portrayed as separate, the case clearly illustrates that structure may influence culture, and culture may influence structure. An example of the former is that innovation became a standing item on the agenda for the weekly unit meeting where all employees participated. As such, it served as a reminder to think about innovation as a part of their ordinary work and also as a symbol that innovation was important. However, such a structural intervention only matters if employees see themselves as active and initiative-taking as above, in an open climate where they all feel trusted by each other and their manager, so structure and culture are intertwined.

Innovation is frequently discussed at unit meetings today, but I think it is because it is something we think about it today. Some years ago, I think such an agenda would have led to silence. (Employee F)

Externally, employees were encouraged to present their innovations during external professional meetings, regional improvement meetings, hospital meetings, etc. There was a continuous flow of presentations in various contexts of the different innovations performed at the unit, and with many different presenters, which supported the external view of the unit as innovative, but also strengthened the employees in seeing themselves as innovative.

Considering how central the former manager was for nurturing the innovation culture at the unit, it is easy to understand the employees' concerns when she decided to leave the unit. However, the culture seems to have persisted even without her. She may have been the force that was needed to create the innovative culture at the unit, but it had become so manifested in structures, values and thinking that the “new” culture endured without her nurturing it. An employee elaborated on what happened after she left:

Of course, we were worried, but we surprised ourselves, I think. Now, it is natural for us to work with innovation projects. We seek solutions, we try. We don’t see it as hard work. We can make a difference … and we have received a lot of attention, which encourage us to continue. (Employee C)

In addition, the successor manager had previously been subordinated to the former manager, having been part of the cultural change to the current innovative culture, which made her determined to keep it that way. She expressed this herself, as did the employees.

She [the current manager] has been part of this process herself, and sees the importance of it. I was never worried that she would work against what we had achieved, and her continuous support for our work with innovation is a proof of that. (Employee A)

Nowadays, in addition to trying to conserve beneficial strategies to support the innovative culture mentioned above, it also manifests in new structures such as in recruitment; where an innovative mind-set and interest in improvement work is a base-criterion when hiring staff and the unit promotes itself as a prominent innovative workplace to attract that kind of employee. The innovative culture is also seen as a positive condition for employee retention, since the employees describe that working in innovation processes is motivating them.

## Discussion

Based on the iceberg model of organisational culture ( Schein, 1990 , 2017 ), we will now take the analysis a step further and then relate the results to previous research. According to the iceberg model, artefacts and espoused values levels are visible, whereas basic assumptions are not visible. The previous result section has described the artefacts level and espoused values level, but the basic assumptions level needs further analysis on the meaning of the visible levels ( Watson, 2006 ). Therefore, in this section we analyse how the visible levels of the organisational culture manifest and represents basic assumptions that were implicitly visible through the empirical description above. Such an analysis also describes the profound shift towards a more innovative culture. However, it may not encompass an exchange of values or basic assumptions, but rather an extension of important values added to the previous ones.

Basic assumptions are often taken for granted and not reflected upon explicitly in a culture ( Schein, 2017 ). Even though they could concern almost everything, assumptions about time and people are often important. In the case above, it is clear that people in the unit have changed their perception of time. They have moved from a strong focus on here-and-now (helping the patient in front of them), which is a strong tendency in healthcare culture ( Grant *et al.* , 2014 ), to also have more of a future perspective (thinking about how to improve the conditions of future patient meetings, etc.). Furthermore, “working time” is no longer seen as something that should be “filled” with “care productions/patient meetings”; employees' need for time to reflect and develop are also considered to enable improve conditions for future care production/patient meetings. Furthermore, the strong individual focus, which is otherwise typical for healthcare ( Exton, 2008 , 2010 ; Grant *et al.* , 2014 ), is complemented here with a view of patient groups, not only individuals, and the groups' needs are the basis for innovations rather than individuals' needs. Lastly, employees seem to have gradually developed a new view of themselves, from being conditioned by external factors (and complaining about them) towards being more active in creating the right conditions, illustrated by the former manager's expression: “It is okay to complain, as long as you are willing to do something about it”. Complaints and frustration then became points of departure for development and innovation, instead of a negative spiral of thought.

Culture is often described as a resource in people's identity work, by providing identity templates that individuals can elaborate to their own individual identity ( Watson, 2008 ). In our case, the decentralised structure and the manager's actions to support employees' innovativeness nurtured the identity template as active and taking initiatives among employees, but this identity template was also enforced by external attention that the unit received. This highlights what previous research has also shown: decentralisation is not only about structure. It is just as much about culture by emphasising certain, values, approaches and principles ( Andersson *et al.* , 2019 ), such as in our case: taking initiative, looking to the future and being responsible not only for performing high-quality patient meetings but also for developing the conditions for future patient meetings. Culture is a process, which means that the values that are central in it must be reified if they are to last ( Smircich, 1983 ). This case shows how organisational structure can support such reification, by continuously pushing for certain themes (as in our case innovation), but also how decentralisation as organisational structure supports and strengthens certain approaches among employees. Organisational structures and organisational cultures are often seen as different and separate from each other, but in practice they are intertwined ( Watson, 2006 ), as exemplified by the current case.

Previous research has emphasised the distribution of resources for innovation ( Exton, 2010 ; Øvretveit *et al.* , 2012 ; Larisch *et al.* , 2016 ), but then mainly in its rational meaning in that it enables possibilities to do things. This is obvious in our case as well, but in terms of culture, distributing resources also has a symbolic meaning ( Watson, 2006 ) because it manifests what is seen as important and what should be prioritised in the organisation. In that sense, distributing internal resources for innovation might have cultural effects that are just as important as structural/rational effects. Alternatively, it should perhaps be stated “in the unit” instead of “in the organisation”, since this culture influenced the unit, but not the hospital as a whole. Upper management's resistance to support the innovations at the unit has equally significant negative symbolical effects, as the priority of innovation in the unit has positive symbolical effects. This resembles research results by Lidman *et al.* (2022) , who find that regarding innovation, first line managers are expected to work according to an exploration logic, while upper management work according to an exploitation logic. The result of this study is also an illustration of Scott *et al* .’s (2003) description of healthcare organisations as consisting of numerous sub-cultures rather than one homogenous organisational culture. Furthermore, it underscores the importance of describing and analysing good examples of innovation cultures ( Exton, 2010 ), such as the present case.

Previous research is ambivalent regarding the role of leadership in innovative cultures in healthcare. First, a lack of leadership is described as one of the main blocking mechanisms for innovation in healthcare ( Larisch *et al.* , 2016 ). Second, it has been emphasised that clinical leaders and managers are central for healthcare innovation, but also that organisational culture works against them in their attempt for innovation ( Exton, 2010 ; Øvretveit *et al.* , 2012 ), which means that managers are seen more as conditioned and delimited by current organisational culture in their innovative efforts. Third, however, Day-Duro *et al.* (2020) presented a more active role of leadership in nurturing innovative culture, highlighting the respectful approach to employees and their values as a way to nurture an innovative organisational culture. In our case, leadership is perhaps the main factor behind nurturing the innovative culture. Our study provides support and more details to Day-Duro *et al.* (2020) , but the difficulties presented by Exton (2010) are also clearly visible. In addition to Day-Duro *et al.* (2020) , leadership is not only about supporting values that employees appreciate, but also about helping employees to think in new ways and to develop new competencies. Furthermore, the decentralised structure is used in the way that Day-Duro *et al.* (2020) suggested – to both empower employees and, in a respectful way, make them accountable for developing and innovating. However, the unit is almost an island, with a different culture, in a sea of a hospital culture that largely has the delimiting values of requirements of conformity that Exton (2010) described as delimiting for healthcare managers.

Even if the current case constitutes a positive example that it is possible to nurture innovative culture in healthcare organisations, it is also clear that this is no easy route. The changing basic assumptions described above illustrate that values and approaches that are important for healthcare in terms of high-quality, patient- and person-centred care, and in that matter has served healthcare organisations well, may be counter-productive in terms of innovation. Consequently, the challenge is to add new values without losing the old ones and to balance these cultural elements carefully (cf. Grant *et al.* , 2014 ).

### Limitations

The study presented here is a single-case qualitative study, which means that transferability is a better concept than generalisability ( Gehman *et al.* , 2018 ). What is important is which boundary conditions make the results transferable to other healthcare setting, or not. The absence of physicians in the unit may have increased the ability of self-governance and nurturing a sub-culture of different character than the hospital at large. Similarly, the homogenous professional structure (almost all were speech therapist) may have made it easier to share values between employees. Furthermore, the fact that the unit had no acute patients made their time easier to plan. These aspects may have created better conditions to nurture an innovative culture than for healthcare units in general. However, one should also recall higher management's lack of interest, or conflicting interest, of requiring performance within the economic frames rather than supporting a development towards a more innovative unit, which can be considered to have worked against nurturing the internal innovativeness and innovative culture. These poorer conditions may be more typical for a healthcare unit. Since Exton (2010) argued that it is important to learn about good innovation examples in healthcare organisations, further research may investigate whether homogenous professional structure and not being an acute unit are important boundary conditions for the results in this study.

## Conclusion

This study contributes to research on innovation in healthcare organisations by describing and analysing how innovative organisational cultures can be nurtured in healthcare organisations. Leadership is shown to be central to supporting values and creating structures that involve employees in new ways of thinking about their work. Furthermore, resources that enable employees to work with innovations are a pre-requisite but also have important signalling effects that influence the culture. An innovative culture may have different characteristics compared to common organisational cultures in healthcare, which tends to be focused on “here-and-now” and individual patients. An innovative culture also means thinking about future conditions and perceiving patients as parts of groups of patients with certain needs. The study emphasised that organisational culture is a process that can be nurtured both by structure and managers' and employees' persistent approaches and principles. It also shows that a decentralised structure is important for an innovative culture.

The study also has implications for practice. It shows that it is possible to nurture an innovative culture at a unit, independent of the characteristics of the culture of the hospital as a whole. Cultural change projects are often over-simplified and based on the inaccurate assumption that culture is something that can be shaped and controlled, which means that most cultural change projects become failures ( Alvesson and Sveningsson, 2015 ). This can lead to the conclusion that culture is impossible to change, which is also inaccurate. The main practical contribution of this research is to pave a middle ground between over-simplified change attempts and being impossible to change. Saying that organisational culture is impossible to control is not the same as saying that it is impossible to influence. However, the road to influence goes through understanding the current culture and then basing these attempts on the understanding of the culture. This paper illustrates how culture can be understood and what cultural aspects could be influenced to a more innovative culture in healthcare. Nurturing innovative culture in healthcare organisations requires managers who committed to development and innovation (rather than conformity), who allow for empowered employees who have development and innovation as parts of their ordinary work. Resources (time and money) are not only important to enable work with innovations, but have important symbolic effects on what should be prioritised in the unit.

There are interesting avenues for further research. Considering the highly institutional character of healthcare organisations ( Reay *et al.* , 2017 ; Andersson and Gadolin, 2020 ; Oborn and Barrett, 2021 ; Oborn *et al.* , 2021 ), which implies that any change in healthcare organisations basically requires an institutional change, it would be interesting to use an institutional logics framework or institutional work framework to understand support for innovation processes in healthcare organisations. In the present paper, the focus is on organisational culture; that is, we seek the explanations within the organisation. However, an institutional framework would allow future research to seek explanations for what happens within the organisation beyond the organisation ( Hinings, 2012 ).
